# Impact of the COVID-19 pandemic on young people with and without pre-existing mental health problems

**DOI:** 10.1038/s41598-023-32918-5

**Published:** 2023-04-14

**Authors:** Ronja Kleine, Artur Galimov, Reiner Hanewinkel, Jennifer Unger, Steve Sussman, Julia Hansen

**Affiliations:** 1 Institute for Therapy and Health Research, IFT-Nord, Harmsstraße 2, 24114 Kiel, Germany; 2 Keck School of Medicine, University of Southern California, 1845 N. Soto Street, Los Angeles, CA 90033 USA

**Keywords:** Psychology, Human behaviour, Risk factors

## Abstract

The objective was to examine pandemic-related changes in depression and anxiety symptoms in adolescents and young adults in Germany considering pre-existing depression and anxiety problems. In this cross-sectional study, 11,523 adolescents and young adults aged 14–21 years who perceived an impact of the Coronavirus disease (COVID-19) pandemic on their mental health reported the frequencies of depression and anxiety symptoms retrospectively for different pre-pandemic and pandemic phases. Data were collected using web-based questionnaires between January 5th and February 20th, 2022. Depression and anxiety were assessed with a modified version of the Patient Health Questionnaire (PHQ-4). Scale-fit cut-offs were used to identify pre-existing elevated depression and anxiety scores. Multilevel mixed linear models were conducted to assess changes in depression and anxiety symptoms from 2019 to 2021 and compare for age, gender and pre-pandemic mental health problems. Among young people who were experiencing mental health changes as a result of the pandemic, the frequency of depression and anxiety symptoms increased during the COVID-19 pandemic. This association was moderated by age, gender, and pre-existing elevated depression/anxiety scores. For young people without elevated pre-pandemic depression/anxiety, the scores increased strongly over time, with 61% reporting elevated depression symptoms and 44% reporting elevated anxiety symptoms in 2021. In contrast, self-perceived change was minimal for adolescents and young adults with elevated pre-pandemic depression and anxiety. Among young people whose mental health has been affected by the COVID-19 pandemic, the group without pre-pandemic mental health conditions reported greater deterioration than those with elevated pre-pandemic depression and anxiety scores. Thus, adolescents and young adults without pre-existing depression and anxiety problems who perceived a change in general mental health due to the pandemic reported an alarming increase in symptoms of depression and anxiety during the COVID-19 pandemic period.

## Introduction

The COVID-19 pandemic has changed the everyday life of people worldwide. For example, the pandemic has led to lockdowns, school closures, and many public area restrictions to control the spread of the virus. A growing body of international literature reports an increase in mental health problems during the COVID-19 pandemic^1–9^. These findings indicate that the COVID-19 pandemic has a negative impact on youth mental health, particularly with symptoms of depression and anxiety. According to a meta-analysis by Racine et al.^6^, the prevalence of depression and anxiety symptoms has doubled since the onset of the pandemic with increasing rates later in the pandemic. Severe restrictions due to lockdown measures, greater limitations of social contacts, and greater perceived changes in life were associated with further mental health impairments^10^. Perceived threat, a potential loss of daily routines, and the loss of positive reinforcers in consequence of restricted social contacts and leisure activities increased the risk of depressive and anxiety symptoms in the COVID-19 pandemic context^11,12^.

Within adult cohorts, younger age and female gender were associated with higher mental health burden^1,13,14^, whereas within cohorts of children and adolescents older age and female gender were associated with impaired mental health^3,4,6,9,15,16^. Besides age and gender, different findings suggest pre-pandemic mental health conditions as another risk factor^1,13,17–21^. Studies observed a higher prevalence of psychopathological symptoms during the pandemic for persons with pre-pandemic mental illness^13,18^ suggesting that this group experienced a greater burden from the pandemic. However, other research suggested that people without pre-pandemic mental health conditions experienced greater deterioration^20^. For example, students with pre-existing mental health concerns showed improving or similar mental health during the pandemic whereas students without pre-existing mental health concerns were more likely to show declining mental health^18^. The authors speculated that the reason for the decline in mental health in groups with no pre-existing mental problems was the response to social isolation as a new, unprecedented situation.

Several German studies found similar results concerning the pandemic’s influence on young persons’ general mental health: during the COVID-19 pandemic, children and adolescents showed continuously increasing emotional problems, a decrease in quality of life, and reported more frequent depression and anxiety symptoms^22–24^. Similarly, German adults reported increased symptoms of anxiety and depression, with over 50% suffering from fear and psychological distress regarding the COVID-19 pandemic during the first lockdown in 2020^25,26^. Again, female and younger adults reported more mental health problems^24,26^.

To our knowledge, only a few studies have assessed pre-pandemic mental health status as a predictor of pandemic-related changes in mental health^18,20^, and none conducted in Germany. Evidence regarding this topic has been restricted to the first stage of the pandemic (May 2020) and adult samples^20^. Therefore, the objectives of the study were to examine the self-reported perceived changes of depression and anxiety symptoms in adolescents and young adults with and without pre-existing problems during the different stages of the COVID-19 pandemic using a retrospective design, considering the influence of age and gender. Based on the findings from previous studies^18,20^ we hypothesized that depression and anxiety symptoms would increase during the pandemic among adolescents without pre-pandemic depression or anxiety, but no particular changes for persons with elevated pre-pandemic depression or anxiety would be observed. We also hypothesized that the female gender and older age would be associated with a greater deterioration.

## Methods

### Study design and sample

Data were derived from a large cross-sectional study on the impact of the COVID-19 pandemic on substance use, media consumption, and mental health among adolescents and young adults in Germany. The data were collected between January 5th and February 20th, 2022 using a web-based questionnaire. Participants were recruited via age-group-specific advertisements on Facebook and Instagram. This method was successfully used in previous studies^27^. By clicking on the link in the advertisements, adolescents and young adults received access to the questionnaire. Information about the survey and protection of data privacy was displayed. Consent was given when continuing the questionnaire.

**Figure 1:**
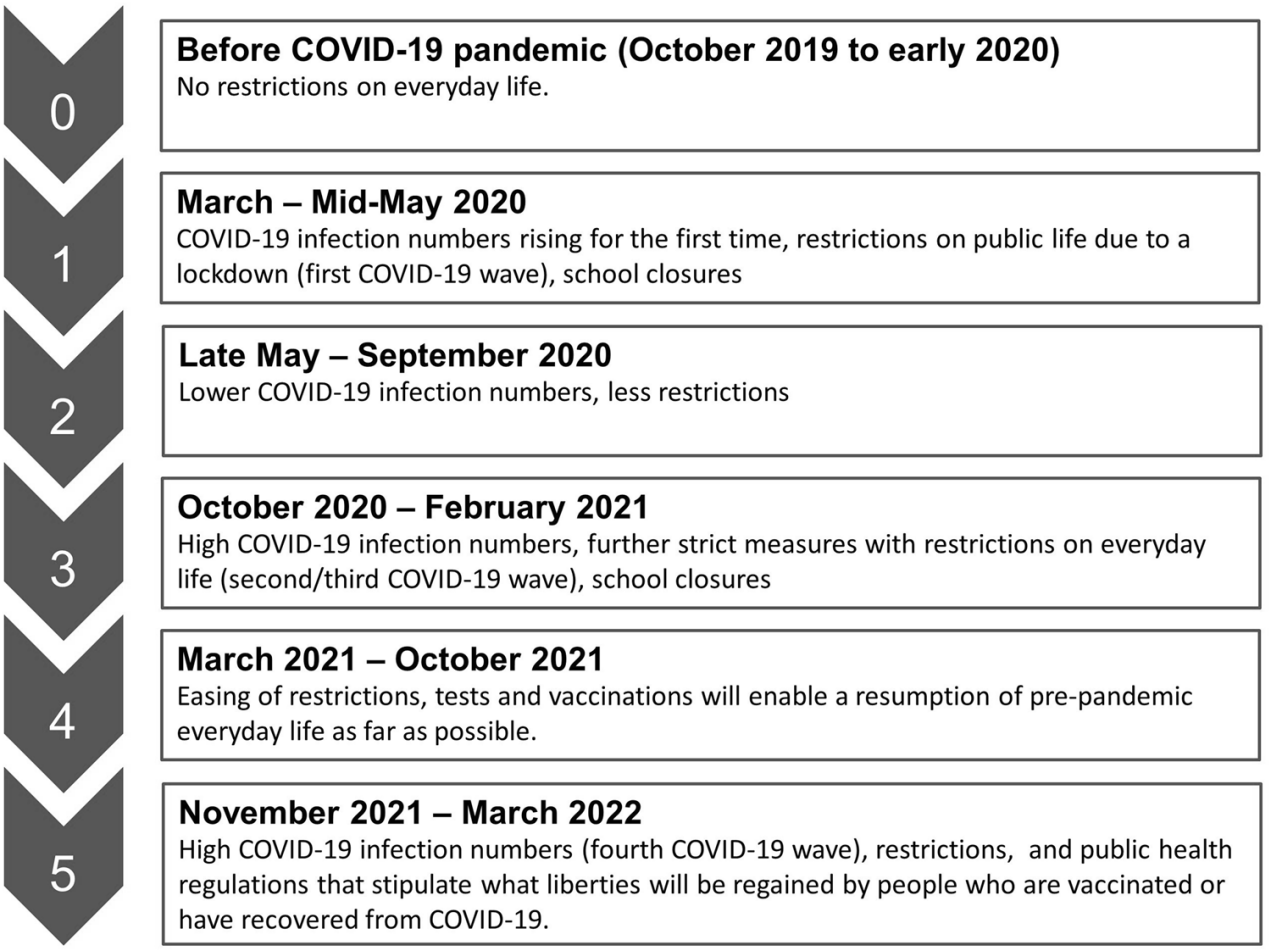
COVID-19 pandemic stages^28^.

The data collection took place when Germany was in the fourth wave of the COVID-19 pandemic, with high restrictions due to high COVID-19 infection cases and public health regulations that stipulate what liberties will be regained by people who are vaccinated or have recovered from COVID-19 (see Fig. 1).

Overall, 14,129 female and male adolescents and young adults provided data on mental health. The inclusion criterion for the main analyses was a pandemic-related change of the general mental health assessed with the following question: *“What would you say, did your psychological wellbeing change as a result of the corona pandemic? That is, whether the pandemic has made you feel different (e.g., happier or sadder, more relaxed or restless)”.* Answering choices were “*No, the pandemic had no impact”, “Yes, I was doing much better than before the pandemic.”, Yes, I was doing better than before the pandemic”, “Yes, I felt worse than before the pandemic”, “Yes, I felt much worse than before the pandemic”.* If a pandemic-related change was indicated (every other answer than *“No, the pandemic had no impact”),* conditional questions about depression and anxiety were asked. In total, N = 11,523 female and male adolescents and young adults aged 14 to 21 answered retrospective questions about depression and anxiety in different periods before and during of the pandemic.

### Measures

*Primary outcomes.* Depressive and anxiety symptoms were assessed using a modified version of the Patient Health Questionnaire (PHQ)-4^29^ (modification: translated to German, adapted to easy language, response scale was modified from a 4-point-Likert-scale to a 21-point slider); (e.g. *“How often have you felt down, depressed, irritable, or hopeless?”*). The PHQ-4 is a widely used questionnaire with good reliability and validity to screen for depression and anxiety symptoms in the general population^29–31^. On a slider from *"not at all"* (0) to *"almost every day"* (20), the participants rated for the periods "2019 (before the corona pandemic)", "2020 (beginning of the corona pandemic)" and "2021 (ongoing corona pandemic)” how often they experienced the described symptoms of depression and anxiety. The scale has been modified to improve recall accuracy, as this can be achieved with broader answering categories^32^. For each of the three periods, a total score was calculated for the depression and anxiety subscales (each subscale with a total score ranging from 0 to 40). Higher scores indicated higher levels of depression or anxiety. The established cut-off^29–31,33^ was transferred to the used answering scale. Scores equal to 20 or above were categorized as elevated depression/anxiety scores. Different studies have shown, that retrospective self-reports of mental health are reliable at the group level^32,34^.

*Sociodemographic correlates/variables.* The survey included questions on age, gender (male coded as 0, female coded as 1), job-related status (student coded as 1, university student coded as 2, trainee as 3, employed et al. as 4, other as 5). Subjective social status was measured with a German translation of the MacArthur Scale, ranging from zero (low income, the worst jobs, the lowest education) to 10 (high income, the best jobs, the highest education)^35,36^.

### Data analyses

Depression and anxiety scores were calculated as described above. According to their indicated value of depression and anxiety symptoms in 2019 (before the pandemic) participants were categorized into two groups: with and without elevated pre-pandemic depression (N = 2309 and N = 9214, respectively) or anxiety (N = 1990 and N = 9533, respectively). Further, two groups were created based on participants’ age: adolescents (14–17 years) and young adults (18–21 years). Only complete cases were analysed.

First, the prevalence of the pandemic-related changes in general health were reported among full sample (N = 14,129). In addition, bivariate comparisons for gender, age and pre-pandemic mental health status were calculated using chi-square-tests.

Second, the prevalence of elevated scores for depression and anxiety before and during the COVID-19 pandemic and the scales’ mean scores were reported for the analytic sample (N = 11,523). In addition, the statistics were also reported for both groups (with vs. without elevated pre-pandemic depression/anxiety scores). Further, bivariate comparisons between the groups were calculated using chi-square-tests, and t-tests.

Third, mixed linear models were set up to estimate the relationship between the outcome depression (and anxiety, respectively) and the Level 1 predictors: COVID-19 periods (categorical), pre-pandemic mental health conditions, age group and gender. Subjective social status and job-related status were added to the model as covariates. Random slopes for individual-specific effects (Level 2) nested within the repeated measures predictor (Level 3) were added. Next, we tested for interactions of all four predictors. Maximum-Likelihood estimation was used, and non-standardized coefficients were calculated. Post-hoc analysis of estimated marginal means (EMMs) was done to examine further relevant differences. Non-standardized beta coefficients (β) and differences between the EMMs with 95% CIs were reported with statistical significance set at *p* < 0.05 (2-tailed). Bonferroni multiple-testing corrections were applied to control the false-discovery rate at 0.05 All statistical analyses were conducted using Stata (SE 17.0)^37^.

### Ethics approval

Ethical approval was obtained from the ethical committee of the German Psychological Society (*Ethikkommission der Deutschen Gesellschaft für Psychologie*, ID: HanewinkelReiner2021-10-21VA).

### Informed consent

All participants within this study provided informed consent. Data protection profile and information have been written in easy language to be understandable for young readers. The survey data was completely anonymous. According to Recital 26 of General Data Protection Regulation (GDPR) “the principals of data protection [including article 8(1)] should […] not apply to anonymous information […]. This Regulation does not therefore concern the processing of such anonymous information, including for statistical or research purposes.” Therefore, no parental consent was needed for adolescents with minimum age of 14 years participating in our anonymous survey.

## Results

### Sample characteristics

The study sample consisted of 6945 (49.2%) adolescents (14–17 years) and 7184 (50.8%) young adults (18–21 years), 48.4% identified as male and 51.6% as female. The average age was 17.7 years (SD = 2.3). Other demographic characteristics are shown in Table 1.

**Table 1:** Participant characteristics for the study sample (N = 14,129).

Variable	N (%) or M (SD)
Age^a^	17.7 (2.3)
Gender	
Male	6844 (48.4%)
Female	7285 (51.6%)
Job-related status	
Students	7400 (52.4%)
University students	2371 (16.8%)
Trainees	2472 (17.5%)
Employed or in volunteering	1235 (8.7%)
Other (unemployed, stay-at-home parent et al.)	651 (4.6%)
Subjective social status^b^	6.1 (1.7)

^a^Age ranged from 14 to 21 years due to inclusion criteria.

^b^MacArthur Scale^35,36^, ranging from 1 (low income, the worst jobs, the lowest education) to 10 (high income, the best jobs, the highest education).

### Pandemic-related change in the general mental health

**Figure 2:**
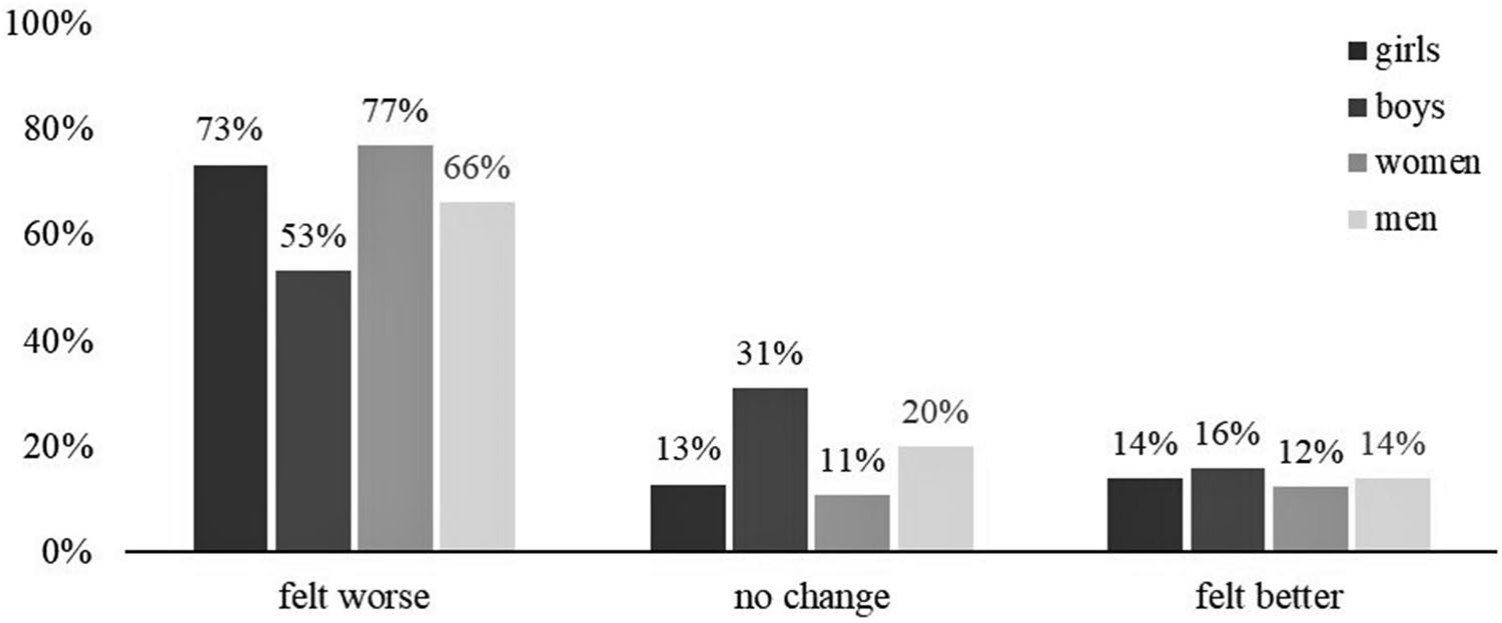
Changes in the general mental health reported by gender (males vs. females) and age group (adolescents vs. young adults) (N = 14,129).

In the study sample (N = 14,129), a pandemic-related change in general mental health was observed in 81.6% of adolescents and young adults. Overall, 13.9% felt better during the pandemic, 67.6% felt worse, and 18.4% reported no change in mental health status. Females and young adults were more likely to report negative changes in mental health compared to males and adolescents (gender: χ^2^ = 504.3, *p* < 0.001; age: χ^2^ = 133.8, *p* < 0.001; see Fig. 2).

Among those reporting a change in general mental health (N = 11,523), individuals with pre-pandemic mental health problems were more likely to report improvement in general mental health due to the COVID-19 pandemic compared to individuals without pre-pandemic mental health problems (depression: 23.6% vs. 15.4%, χ^2^ = 86.8, *p* < 0.001; anxiety: 23.7% vs. 15.7%, χ^2^ = 73.8, *p* < 0.001).

### Changes in depression symptoms

Descriptive analysis showed increasing numbers of depression symptoms during the COVID-19 pandemic with 20.0% reporting elevated depression symptoms before the pandemic, 43.9% in the first (2020) and 63.4% in the second year (2021) of the pandemic (see Table 2).

**Table 2:** Parameters of the PHQ-4 depression and anxiety subscales for different periods before and during the COVID-19 pandemic (N = 11,523) in both groups (with vs. without pre-pandemic depression/anxiety).

Period	Total		With elevated pre-pandemic score		Without elevated pre-pandemic score	
	mean (SD)	No. (%) elevated score^a^	Mean (SD)	No. (%) elevated score^a^	Mean (SD)	No. (%) elevated score^a^
	Depression					
2019	11.1 (9.7)	2309 (20.0%)	26.9 (6.4)	2309 (100%)	7.1 (5.4)	0 (0%)
2020	18.8 (10.3)	5063 (43.9%)	26.2 (9.4)	1776 (76.9%)	16.9 (9.6)	3287 (35.7%)
2021	23.7 (10.8)	7306 (63.4%)	26.2 (10.6)	1702 (73.7%)	23.1 (10.8)	5604 (60.8%)
	Anxiety					
2019	10.3 (10.0)	1990 (17.3%)	28.7 (6.7)	1990 (100%)	6.4 (5.1)	0 (0%)
2020	16.8 (11.4)	4366 (37.9%)	28.8 (9.2)	1683 (84.6%)	14.3 (10.1)	2683 (28.1%)
2021	20.4 (12.2)	5804 (50.4%)	29.2 (10.1)	1629 (81.9%)	18.6 (11.8)	4175 (43.8%)

All bivariate comparisons between both groups are significant at level *p* < .001.

^a^Scores equal to 20 or above were categorized as elevated scores in depression/anxiety.

The cut-off at 20 is based on an established cut-off score applied to the modified response scale.

The mixed model predicting depression scores (N = 11,523) revealed a significant main effect of time (β_2020_ = 7.7, 95% CI: 7.5–7.9; β_2021_ = 12.6, 95% CI: 12.4–12.9), controlling for all other predictors. Depression scores grew significantly over time, EMMs in 2019: 11.1 (95% CI: 11.0–11.2), 2020: 18.8 (95% CI: 18.6–19.0), 2021: 23.7 (95% CI: 23.5–23.9). Interactions of all predictors with time were significant at *p* < 0.001. Contrasts for two-way-interactions are presented in Table 3. Females reported higher depression scores compared to males for each pandemic period with increasing differences over time. No differences were found between the age groups in 2019, but with the pandemic’s onset young adults reported increasingly higher depression scores compared to adolescents. Among participants without elevated pre-pandemic depression symptoms (N = 9214) the depression scores increased strongly over time, EMMs in 2019: 7.2 (95% CI: 7.0–7.3), 2020: 17.0 (95% CI: 16.8–17.2), 2021: 23.2 (95% CI: 23.0–23.4). In contrast, for those with elevated pre-pandemic depression (N = 2309) the scores decreased marginally in the first pandemic year and then stayed stable in the second year, EMMs 2019: 26.7 (95% CI: 26.5–26.9), 2020: 25.9 (95% CI: 25.6–26.3), 2021: 25.8 (95% CI: 25.4–26.3).

**Table 3:** Post-hoc calculated contrasts within the linear mixed model (N = 11,523) for depression and anxiety scores with pandemic period, gender, age group and pre-pandemic depression/anxiety status as fixed effects and individual-specific random effects nested within the time variable, adjusted for job-related status and subjective social status.

Contrast	Difference (95% CI)	
	Depression	Anxiety
Period of time		
2020 versus 2019	7.7 (7.5, 7.9)***	6.5 (6.3, 6.7)***
2021 versus 2020	4.9 (4.6. 5.2)***	3.6 (3.3, 3.9)***
Gender × period		
2019 (female vs. male)	0.5 (0.2, 0.8)***	1.3 (1.0, 1.5)***
2020 (female vs. male)	2.1 (1.7, 2.5)***	3.4 (3.0, 3.8)***
2021 (female vs. male)	3.7 (3.2, 4.2)***	5.3 (4.8, 5.7)***
Age group × period		
2019 (adult vs. adolescent)	0.3 (− 0.1, 0.6)	0.7 (0.3, 1.0)***
2020 (adult vs. adolescent)	0.8 (0.3, 1.3)***	1.4 (0.9, 1.9)***
2021 (adult vs. adolescent)	1.1 (0.5, 1.6)***	1.0 (0.5, 1.6)***
Pre-pandemic depression × period		
2019 (with vs. without)	19.5 (19.2, 19.8)***	22.0 (21.6, 22.3)***
2020 (with vs. without)	8.9 (8.4, 9.4)***	13.9 (13.4, 14.5)***
2021 (with vs. without)	2.7 (2.1, 3.3)***	9.9 (9.2, 10.5)***
With (2020 vs. 2019)	− 0.9 (− 1.4, − 0.4)***	− 0.2 (− 0.7, 0.3)
With (2021 vs. 2020)	− 0.2 (− 0.8, 0.5)	0.3 (− 0.5, 1.0)
Without (2020 vs. 2019)	9.7 (9.5, 10.0)***	7.9 (7.7, 8.1)***
Without (2021 vs. 2020)	6.1 (5.7, 6.4)***	4.3 (4.0, 4.6)***

*p* values are Bonferroni-corrected. * *p* < .05, ** *p* < .01, *** *p* < .001.

**Figure 3:**
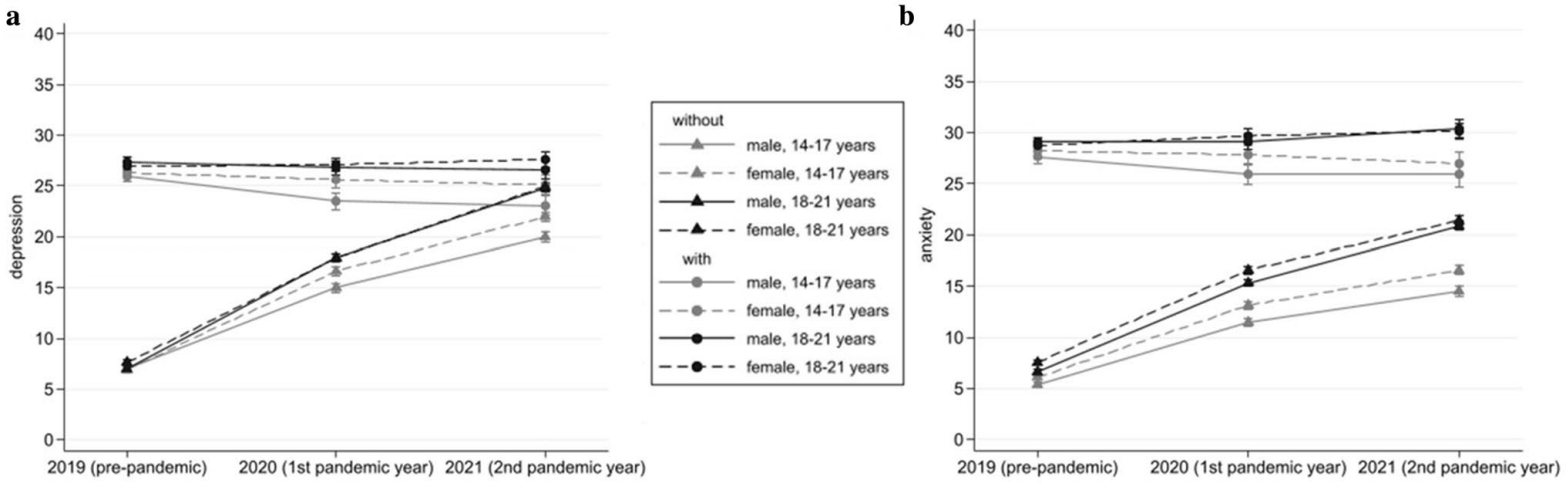
Estimated marginal means and 95% CIs on the depression (**a**) and anxiety (**b**) subscale across time, gender, age groups and pre-pandemic depression/anxiety status (with vs. without elevated symptoms) (N = 11,523).

EMMs and 95% CIs for the four-way interaction of time with age group, gender, and pre-pandemic depression status are shown in Fig. 3. Average depression scores of females (both age groups) and male young adults without elevated pre-pandemic depression passed the applied scale-fit 20-point-cut-off in 2021, EMMs for 18–21-year-old males: 22.0 (95% CI: 21.5–22.4), 14–17-year-old females: 24.8 (95% CI: 24.4–25.3), 18–21-year-old females: 24.9 (95% CI: 24.5–25.4). For male adolescents without elevated pre-pandemic depression, the average depression score in 2021 was not significantly different from the 20-point-cut-off, EMM = 20.0 (95% CI: 19.5–20.5).

Among participants with elevated pre-pandemic depression symptoms, the depression scores decreased in 2020 compared to 2019 for male adolescents only (diff =  − 2.5, 95% CI: − 3.8 to − 1.1), but not for the other three age/gender groups. From 2020 to 2021 the depression scores remained stable. Overall respondents with elevated pre-pandemic depression symptoms had significantly higher depression scores than those without pre-pandemic depression but with diminishing differences over time, the difference in 2021 for 14–17-year-old males: 3.1 (95% CI: 1.5–4.7), 14–17-year-old females: 1.7 (95% CI: 0.4–3.1), 18–21-year-old males: 3.1 (95% CI: 1.6–4.7), 18–21-year-old females: 2.7 (95% CI: 1.4–3.9).

### Changes in anxiety symptoms

Regarding anxiety, increasing numbers of symptoms were observed during the pandemic with 17.8% reporting elevated symptoms in 2019, 38.4% in the first, and 51.1% in the second year of the COVID-19 pandemic (see Table 2).

The mixed model predicting anxiety scores (N = 11,523) revealed a significant main effect of time (β_2020_ = 6.5, 95% CI: 6.3–6.7; β_2021_ = 10.1, 95% CI: 9.9–10.3), controlling for all other predictors. Anxiety scores grew significantly over time, EMMs in 2019: 10.3 (95% CI: 10.2–10.4), 2020: 16.8 (95% CI: 16.6–17.0), 2021: 20.4 (95% CI: 20.2–20.6). Interactions of all predictors with time were significant at *p* < 0.001. Contrasts for two-way interactions are presented in Table 3. Females reported higher anxiety scores compared to males with increasing differences over time. Young adults reported marginally higher pre-pandemic anxiety scores compared to adolescents and increasingly higher scores during the pandemic. Among participants without elevated pre-pandemic anxiety symptoms (N = 9533) the anxiety scores increased strongly over time, EMMs in 2019: 6.5 (95% CI: 6.4–6.6), 2020: 14.4 (95% CI: 14.2–14.6), 2021: 18.7 (95% CI: 18.5–18.9). In contrast, for those with elevated pre-pandemic anxiety (N = 1990) the scores stayed stable during the pandemic, EMMs 2019: 28.5 (95% CI: 28.2–28.7), 2020: 28.3 (95% CI: 27.9–28.7), 2021: 28.6 (95% CI: 28.1–29.1).

EMMs and 95% CI for the four-way-interaction of time with age group, gender, and pre-pandemic anxiety status are shown in Fig. 3. Average anxiety scores for females (both age groups) without elevated pre-pandemic anxiety status passed the applied scale-fit 20-point-cut-off in 2021, while it remained significantly below cut-off points for males (both age groups); EMMs for 14–17-year-old males: 14.5 (95% CI: 14.1–15.0), 14–17-year-old females: 20.9 (95% CI: 20.5–21.3), 18–21 year-old males: 16.6 (95% CI: 16.1–17.0), 18–21-year-old females: 21.4 (95% CI: 21.0–21.8). In the group with elevated pre-pandemic anxiety symptoms the anxiety scores for male adolescents decreased in 2020 compared to 2019 (diff =  − 1.7, 95% CI: − 3.2 to − 0.3), while the scores increased marginally for female young adults (diff = 0.9, 95% CI: 0.1–1.9) and remained unchanged for female adolescents and male young adults. From 2020 to 2021 the scores remained stable. Over all periods of time respondents with elevated pre-pandemic anxiety symptoms had significantly higher anxiety scores compared to those without pre-pandemic anxiety but with diminishing differences over time, the difference in 2021 for 14–17-year-old males: 11.4 (95% CI: 9.5–13.3), 14–17-year-old females: 9.5 (95% CI: 8.0–11.0), 18–21-year-old males: 10.4 (95% CI: 8.7–12.2), 18–21-year-old females: 8.8 (95% CI: 7.5–10.0).

## Discussion

### Key findings

In this cross-sectional study with 14,129 adolescents and young adults aged 14 to 21 years from Germany, the majority (81.6%) reported a COVID-19 pandemic-related change in general mental health. For those who perceived a COVID-19 pandemic-related change in general mental health, different trajectories for individuals with and without pre-pandemic elevated depression and anxiety symptoms were observed. In contrast to those with elevated pre-pandemic depression and anxiety scores, adolescents and young adults without pre-existing depression and anxiety problems reported an alarming increase in symptoms of depression and anxiety during the COVID-19 pandemic.

### Limitations

There are several limitations of this study that must be considered when interpreting the results below. The cross-sectional design limits the ability to infer causality. Depression and anxiety symptoms were assessed retrospectively for different periods in the last three years instead of using a longitudinal design with repeated measures, resulting in subjective data and the risk of recall and memory biases. However, past research has shown that subjective mental health data elicited using retrospective questions are consistent at the aggregate level^32^. To reduce recall and memory biases, introductory texts or summary questions as well as specific anchor points can help respondents to remember their psychological health status^32^. The onset of the pandemic is a very remarkable anchor point. Further, short texts were used to help respondents to recall the different periods. Asking broader questions and offering broader answering categories may also help to increase recall accuracy^32^. For this reason, we used a wider scale and sliders instead of a 4-point Likert scale.

Depression and anxiety were assessed using only two-item subscales, but with good validity in the original version^30,31,33^. The scales serve as screening instruments, only assessing major symptoms of the underlying mental disorders. Furthermore, the wording of the items was adapted to simple language and the response scale was modified from a 4-point-Likert-scale to a 21-point slider. This new scale has not been validated. The reasons for the modification lie in the retrospective nature of the questions. The use of an indirect measure method does not ensure that reported changes are caused only by the COVID-19 pandemic. It does not control for confounding critical life events. However, the results are consistent with reported changes assessed with the direct measure examining the impact of the pandemic on mental health. Furthermore, we had to use filter questions in the questionnaire due to economic reasons and only asked those who indicated that their mental health was affected by the pandemic in more detail about depression and anxiety. Hence, the reported depression and anxiety levels only include those who are vulnerable to their general mental health being affected by the pandemic situation. Therefore, the levels of depression and anxiety were not analysed in the group of adolescents and young adults who experienced no pandemic-related changes in their general mental health—however, this only comprises a small group with 18% of the total sample. Individuals who did not perceive any changes should report stable depression and anxiety scores over the pandemic phases. Nevertheless, it remains unanswered in the present study whether adolescents and young adults who have not experienced pandemic-related changes had pre-pandemic elevated anxiety and depression scores or not. Hence, the results can only be generalized to the subpopulation that self-perceived a pandemic-related change in their mental health.

Finally, the PHQ-4 should not be used for diagnostic classification, it serves as a screening tool in the sense of a "first step" approach. Therefore, prevalence estimates for adolescents and young adults with depression/anxiety disorders cannot be derived from our findings. Rather, the results are estimates for those who are at an increased risk of having depression or anxiety disorders. However, further diagnostics are urgently required for the group at risk.

### Interpretation

The increase in depression and anxiety which we have found in the majority of those adolescents and young adults, who experienced a COVID-19 pandemic-related impact on their general mental health, has also been observed in other countries^3,38,39^. We found that the changes were larger for depression symptoms than for anxiety ones. Six out of ten met the screening criteria for depression in 2021, which is three times the size compared to what it was before the COVID-19 pandemic. Five out of ten met the screening criteria for anxiety in 2021, compared to less than two in 2019. Loss of peer interactions, ongoing social isolation, financial difficulties, missed milestones, restricted leisure activities and school disruptions are just some burdens the pandemic and the infection control measures put on adolescents and young adults, being possibly responsible for the negative changes in general mental health since the pandemic’s beginning. As those restrictions accumulated during the ongoing pandemic, the mental burden got worse resulting in further aggravation of depression and anxiety symptoms over time. Ravens-Sieberer and colleagues^24^ found similar higher mental health burdens in the later waves of the pandemic compared to the first month after the pandemic’s onset. Racine and colleagues also observed increasing rates of depression and anxiety later in the pandemic^6^.

Social media use played an important role during the COVID-19 pandemic as it provided an opportunity to stay in touch with friends and family, thereby reducing negative mental health effects such as loneliness, anxiety, and depression^40^. Not only has social media use increased, but virtual conferencing became ubiquitous in the lives of people of all ages. Even though social media and video-conferencing platforms supported human interactions in times with strict lockdown measures, the increased use may be linked with other mental health issues, such as body dysmorphia due to constant access to one’s appearance^41^. Further advantages and disadvantages of social media use in the context of COVID-19 are discussed by Sarangi et al.^40^.

Gender is an important predictor for pandemic-related changes in depression and anxiety. Regarding risk factors for negative changes, findings indicate that females are more vulnerable to the adverse effect of the COVID-19 pandemic on their mental health. This finding is supported by other studies^3,17,38^. Age also had a significant but smaller effect with young adults showing more symptoms of depression and anxiety compared to adolescents with diverging trajectories over the pandemic. However, the female gender as well as older age within the observed age groups were already associated with higher levels of anxiety and depression before the COVID-19 pandemic^42,43^. In addition, different groups are disproportionally affected by the pandemic restrictions due to other life events; for instance, young adults graduating from school and moving out of town during the COVID-19 pandemic may have limited opportunities to meet new people or may have limited ability to spend a year abroad. Different studies observed that increased feelings of loneliness within groups of adolescents and young adults were strongly associated with higher levels of depression and anxiety during the pandemic^9,13,16,44^. Furthermore, Tetreault et al. stated that more disruptions in daily life as well as school and extracurricular activities were associated with increased symptoms of anxiety and depression^4^. Explaining the gender differences, Thorisdottir et al.^3^ argue that females have an increased sensitivity to interpersonal stressors due to hormonal influences and are more likely to engage in social behaviour to cope with stress which was not possible during the pandemic^45,46^. This assertion is supported by the finding, that females reported greater changes in loneliness compared to males^9^

The most important differences were found regarding the pre-pandemic mental state. Although a pandemic-related change in general mental health was reported by all adolescents and young adults in the analysis sample, those with elevated pre-pandemic depression or anxiety scores reported no or only marginal changes during the COVID-19 pandemic. On the other hand, alarming changes were reported by those who had no elevated depression or anxiety symptoms before the onset of the COVID-19 pandemic. They reported a significant increase in depression as well as anxiety in the first pandemic year and ongoing aggravation in the second year. One reason for this phenomenon could be the presence of a measurement limitation called the ceiling effect. However, this is contradicted by the observation that the estimated marginal means in the group with pre-existing mental health problems were well below the upper limit of the scale. Moreover, about one-fourth of participants in the group with pre-existing mental problems reported feeling better because of the pandemic, compared to only 15–16% in the group without pre-existing mental problems. Few other studies analysed the impact of pre-pandemic mental health and reported similar findings^17,18^. Persons with pre-pandemic mental health conditions still reported higher levels of symptoms during the pandemic but those without pre-pandemic mental health conditions reported greater deterioration. As reported by Hamza et al., one possible reason may be that feelings of social isolation and loneliness are associated with greater mental health symptoms^18^. Persons without pre-existing mental health concerns reported increased social isolation and loneliness while those with pre-pandemic mental health concerns reported no change. The authors argue that this second group already felt socially isolated and lonely before the pandemic. One further assumption might be that persons with pre-pandemic depression or anxiety disorder experienced short-term symptom relief because of the avoiding character of the staying-at-home guidelines^47^.

### Implications

Adolescents and young adults are at high risk of being negatively mentally affected by the COVID-19 pandemic especially those without pre-pandemic elevated depression and anxiety. It is possible, that individuals will show improving mental health with the proceeding relaxation of the pandemic and accompanying measures. Society should give young people the chance to resume normal everyday life and to catch up on missed opportunities from the last two years. Concerning the other part of adolescents and young adults having difficulties improving their mental health, the public health system should offer low-threshold help for this vulnerable group to deal with the impact of the pandemic and to acquire functional coping strategies. Possibly, school-based mental health professional work should be expanded both online and in-person to help youth process the negative impact of the pandemic and improve coping skills. Teachers and parents should be sensitised to recognize and address mental health problems. Additionally, low-threshold offers should also be established in other institutions such as universities to reach young adults after school. There is also a need for national efforts, involving health authorities, to ease the stigma surrounding mental illness^48^.

Further research should concentrate on follow-up measurements to monitor future developments, especially when the pandemic no longer dictates everyday life, and identify young people that show ongoing high levels of depression and/or anxiety to offer specific help.

## Data Availability

The datasets used and/or analysed during the current study are available from the corresponding author on reasonable request.

## Abbreviations

PHQ: Patient Health Questionnaire
COVID-19: Coronavirus disease 2019
EMMs: Estimated marginal means
CI: Confidence interval
